# Radiation Resistant Vanadium-Graphene Nanolayered Composite

**DOI:** 10.1038/srep24785

**Published:** 2016-04-21

**Authors:** Youbin Kim, Jinwook Baek, Sunghwan Kim, Sangmin Kim, Seunghwa Ryu, Seokwoo Jeon, Seung Min Han

**Affiliations:** 1 Graduate School of Energy, Environment, Water and Sustainability, Korea Advanced Institute of Science & Technology, Daejeon 305-338, Korea; 2 Department of Materials Science and Engineering and Graphene Research Center of KI for the NanoCentury, Korea Advanced Institute of Science & Technology, Daejeon 305-338, Korea; 3 Department of Mechanical Engineering, Korea Advanced Institute of Science & Technology, Daejeon 305-338, Korea

## Abstract

Ultra high strength V-graphene nanolayers were developed for the first time that was demonstrated to have an excellent radiation tolerance as revealed by the He^+^ irradiation study. Radiation induced hardening, evaluated via nanopillar compressions before and after He^+^ irradiation, is significantly reduced with the inclusion of graphene layers; the flow stresses of V-graphene nanolayers with 110 nm repeat layer spacing showed an increase of 25% while pure V showed an increase of 88% after He^+^ dosage of 13.5 dpa. The molecular dynamics simulations confirmed that the graphene interface can spontaneously absorb the nearby crystalline defects that are produced from a collision cascade, thereby enhancing the lifetime of the V-graphene nanolayers via this self-healing effect. In addition, the impermeability of He gas through the graphene resulted in suppression of He bubble agglomerations that in turn reduced embrittlement. *In-situ* SEM compression also showed the ability of graphene to hinder crack propagation that suppressed the failure.

Advanced structural material for next generation nuclear facilities require enhanced radiation resistance under harsh irradiation environments to ensure an extended lifetime and therefore the safety of nuclear energy systems^1^. For example, structural materials for fusion and fission reactors need to withstand the damage arising from a high radiation dose of ~100 dpa^2^,^3^ while also maintaining its high strength at elevated temperatures. Vacancies, interstitials, Frenkel defects, vacancy clusters or voids are examples of crystalline defects that are produced as a result of displacement cascade under high-energy neutron radiation environments^4^,^5^. In addition, He gas is a transmutation product of the neutron irradiation process^6^, but the low solubility of He results in precipitation and growth of He bubbles^7^. Accrual of such defects can lead to radiation induced mechanical hardening and embrittlement that can cause intergranular failure of the structural material. Hence, development of new material with enhanced radiation tolerance that can retain its mechanical properties under harsh conditions put forth by the nuclear reactors is of critical importance.

Current approaches in enhancing the radiation tolerance include selection of material with reduced scattering cross-section for reduced swelling and engineering of its microstructure to reduce the radiation induced crystalline defects. There are limited metal choices available for nuclear structural applications, and steel is the most widely used material in nuclear facilities. Ni-based alloys are used in applications requiring stability at high temperatures, while Vanadium (V)-based alloys are known to have lower neutron cross section, and reduced radiation induced swelling, and moderate high temperature strength^8^,^9^,^10^,^11^,^12^. Although only limited choices are available for the metal selection, further engineering of its microstructure can significantly enhance the radiation resistance. For example, it is widely accepted that grain boundaries and interfaces can act as sinks for the radiation induced crystalline defects that are produced from displacement cascade under high energy irradiation^13^. Therefore, inclusion of high density of either homogeneous interfaces (*i.e.* grain boundary) or heterogeneous (secondary phases) can significantly enhance the radiation tolerance and has been actively researched over the last few decades. One example of using both homogeneous and heterogeneous interfaces in bulk alloys is polycrystalline ferritic/martensitic (F/M) steels with oxide-dispersions (ODS), which is regarded as an effective nuclear structural material due to the ability to absorb crystalline defects at the grain boundaries (*i.e.* small grain sizes) as well as the interfaces formed with the oxide phases that are acting as sinks for radiation induced crystalline defects^14^,^15^,^16^,^17^.

A particularly interesting class of material with enhanced radiation resistance is the metallic nanolayered composite that has been reported to have ultra-high strength^18^,^19^,^20^,^21^ and radiation resistance^22^,^23^,^24^ due to the extremely high density of heterophase interfaces when the repeat layer spacing is reduced down to the nanoscale. The strengthening mechanism in nanolayered structure is due to the effective constraint of the dislocation movements by the high density of interfaces^25^,^26^. The well-known Hall-Petch effect^27^ governs the strengthening mechanism in metallic nanolayered composites with repeat layer spacing down to few tens of nanometers, where 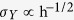. Here, 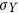 is the yield strength and h is the repeat layer thickness. In addition, atomic-scale simulations using embedded atom (EAM) potentials of Cu-Nb nanolayered composite have shown that the point defects that are produced from a displacement cascade can be spontaneously absorbed or self-healed by the interfaces of Cu-Nb nanolayers that resulted in lower defect density in comparison to that of pure Cu or Nb^24^,^28^,^29^,^30^. In another study by Fu *et al.*^22^,^23^, the helium bubble aggregations were suppressed by the interfaces in Cu-V nanolayers, and the magnitude of radiation hardening and swelling was confirmed to be significantly lower in Cu-V nanolayers as the repeat layer spacing is reduced from 50 nm to 2.5 nm.

In our previous work, we reported that graphene with its 2D geometry, outstanding mechanical properties of 130 GPa in strength and Young’s modulus of 1 TPa can be an effective strength enhancer when incorporated in the form of metal-graphene nanolayered composite^31^,^32^. The strength of Ni-graphene nanolyered composite with 100 nm repeat layer spacing was 4.0 GPa, which is over 50% of the theoretical strength of Ni. Graphene is also expected to be excellent in enhancing the radiation resistance since graphene is impermeable to all standard gases, including He gas and failure due to He bubble agglomerations in the metals could be prevented^33^,^34^,^35^. In addition, the metal-graphene interface can potentially have self-healing ability to absorb the point defects that are produced during high energy radiation environments. In this work, we developed radiation resistant V-graphene nanolayered composite for the first time, and analyzed its self-healing and He bubble suppression capabilities by using nanopillar compression tests before and after the He^+^ irradiation. To further understand the deformation mechanism of V-graphene nanolayers after ion irradiation, *in-situ* SEM nanopillar compression testing, transmission electron microscopy (TEM) microstructure analysis of the irradiated specimens, and molecular dynamics simulations were performed to understand the enhanced radiation resistance.

## Results

### Strengthening effect in V-graphene nanolayers

**Figure 1:**
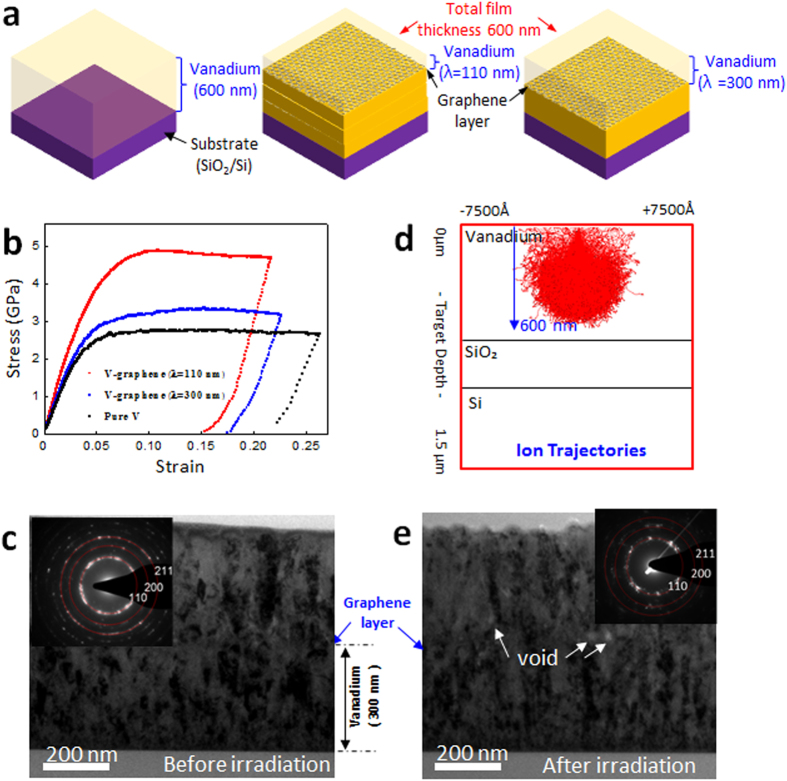
(**a**) Schematic for pure V and V-graphene nanolayers with repeat layer spacing (λ) of 110 nm and 300 nm. (**b**) Stress-strain curve determined from nanopillar compression testing of pure V, and V-graphene nanolayers with 110 nm and 300 nm repeated layer spacings. (**c**) TEM image showing the nanocrystalline nature of V-graphene with 300 nm repeated layer spacing. (**d**) SRIM ion trajectories of He^+^ irradiation on V thin film under condition of 120 keV. (**e**) TEM image showing the radiation induced grain growth after He^+^ irradiation.

V-graphene nanolayers with 110 nm and 300 nm repeat layer spacings as well as pure V thin film were prepared as outlined in the schematic in Fig. 1a. Nanopillars were synthesized from pure V, V-graphene nanolayers and tested in compression as shown in Fig. 1b. The stress-strain response is indicative of a clear strength enhancement with the inclusion of graphene layers, and the strength is increased further for finer repeat layer spacings. Pure V and V-graphene nanolayers with 300 nm and 110 nm repeat layer spacings showed an average flow stress at 5% plastic strain of 2.5 GPa, 3.1 GPa, and 4.8 GPa, respectively. The strengthening effect of a single atomic layer thickness graphene was previously reported to be due to effective constraint on the dislocation motion across the interface^31^. Similar strengthening effect is expected to be responsible for those V-graphene nanolayers, although the V-graphene may have further contributions arising from smaller grain sizes in comparison to the previously reported Cu, Ni-graphene nanolayers.

The synthesized V-graphene nanolayers with 300 nm repeat layer spacing were analyzed for the initial microstructure using the TEM. Both the micrograph and the selected area diffraction (SAD) shown in Fig. 1c indicate that the V layers have grain size in the range of tens of nanometers, which was significantly smaller than the grain sizes of Cu or Ni-graphene nanolayers reported previously^31^. The smaller grain size may be due to higher melting point and therefore lowers diffusivity of V which hinders growth of larger grains during sputtering^36^. The V layers had columnar grain structure with preferable out-of-plane texture of (111), but the layers were crystallographically mismatched in-plane due to the presence of the graphene. The resulting microstructure of nanocrystalline grains with the presence of planar graphene interfaces is expected to be highly efficient in absorbing crystalline defects that are generated during the radiation process. Since the graphene layer is transferred onto V thin film at room temperature, the vanadium carbide (VC) did not form as evidenced by X-ray photoelectron spectroscopy (XPS) data shown in the Supplementary Information Fig. S1.

### Radiation tolerance of V-graphene nanolayers

To analyze the radiation tolerance of V-graphene nanolayers, He^+^ implantation at 120 KeV was used to irradiate pure V, and V-graphene with different repeat layer spacings. The irradiation condition was simulated using Stopping Range of Ions in Material (SRIM)^37^ assuming that the specimen was pure V (see Fig. 1d), and the calculations indicate that He^+^ ions have sufficient energy to reach down close to the substrate interface (with the penetration depth of ~600 nm on vanadium thin film), thereby fully irradiating the V layers that are to be tested for mechanical properties. The irradiated specimens were analyzed for the presence of crystalline defects and any changes in the microstructure, and detailed cross-sectional TEM image with its SAD patterns with irradiated V-graphene nanolayers with 300 nm repeat layer spacing are shown in Fig. 1e. Voids are observed along the graphene interface and at the grain boundaries, and the damage is more pronounced in the top V layer than in the V layer below the graphene interface. In addition, the SAD pattern from after irradiation indicates that the radiation induced grain growth is more observable, where discrete diffraction spots start to appear from the previously recorded diffraction ring. The cause for such radiation induced grain growth is well documented, where the grain boundary migration occurs by irradiation induced thermal spike^38^,^39^. Although such radiation induced grain growth can reduce the embrittlement by the He concentration at grain boundaries, the observation of such phenomena is already indicative of heavy damage being present in the irradiated metal.

**Figure 2:**
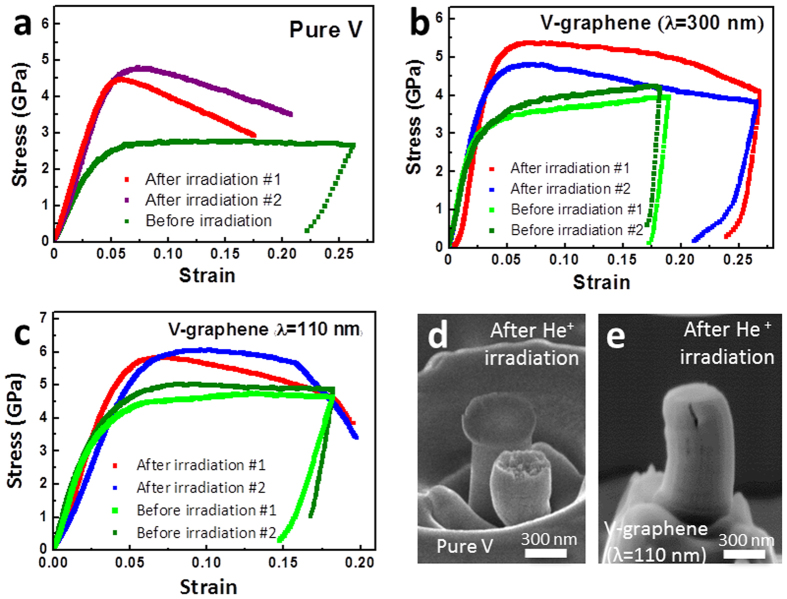
Nanopillar compression tests before and after He^+^ irradiation. Stress-strain curves for (**a**) pure V, V-graphene with repeat layer spacing of (**b**) 300 nm, (**c**) 110 nm. SEM images of nanopillars after compression testing for He^+^ irradiated (**d**) pure V, and (**e**) V-graphene with 110 nm repeat layer spacing indicating that the crack propagation was suppressed by the graphene interface. Full *in-situ* SEM compression movie for irradiated V-graphene with 110 nm repeat layer spacing is available at Supplementary Movie 1.

In the presence of radiation induced defects and He bubble formations, metals can become harder and more brittle, as known as radiation induced hardening. The He^+^ irradiated pure V and V-graphene nanopillars were then evaluated for radiation induced hardening by comparing the nanopillar compression results before and after the irradiation as shown in Fig. 2a,c. The irradiated samples commonly showed an increase in strength and more pronounced brittle failure in comparison to the non-irradiated specimens. For the case of pure V, irradiation nanopillar resulted in significant hardening and brittle failure presumably due to formation of crystalline point defects that can agglomerate to form voids, which agrees favorably with previous report from Masahiro *et al.* for the case of bulk V upon irradiation^40^. According to the Masahiro *et al.*, the He doped bulk V showed hardening of up to twice the original strength and the corresponding brittle failure was reported, where the ductility was reduced from 26% to 4%. For the case of V-graphene nanolayers, however, a significantly smaller degree of embrittlement and radiation induced hardening was observed with the inclusion of graphene, especially with smaller repeat layer spacings or higher density of graphene layers. The radiation induced hardening resulted in increase in flow stress at 5% plastic strain from 4.8 GPa to 6 GPa (25% increase) for the V-graphene with 110 nm repeat layer spacing, from 3.1 GPa to 5.0 GPa (61% increase) for the V-graphene with 300 nm, and from 2.5 GPa to 4.8 GPa (88% increase) for the case of pure V.

*In-situ* SEM nanopillar compression tests were performed for direct observations of the deformation process for irradiated pure V and V-graphene nanolayers with 110 nm repeat layer spacing. For the case of irradiated pure V, brittle failure was observed at 20% strain as shown in Fig. 2d. In contrast, the V-graphene with 110 nm repeat layer spacing also started with a crack initiation at the top most V layer, but the crack did not propagate to the lower V layer as shown in Fig. 2e and in the Supplementary Movie 1. The crack growth was hindered by the graphene interface, and thereby suppressing the brittle failure that resulted in the V-graphene nanopillar being able to withstand strain of up to 20% without failure.

## Discussion

### Suppression of He bubbles at the graphene interface

**Figure 3:**
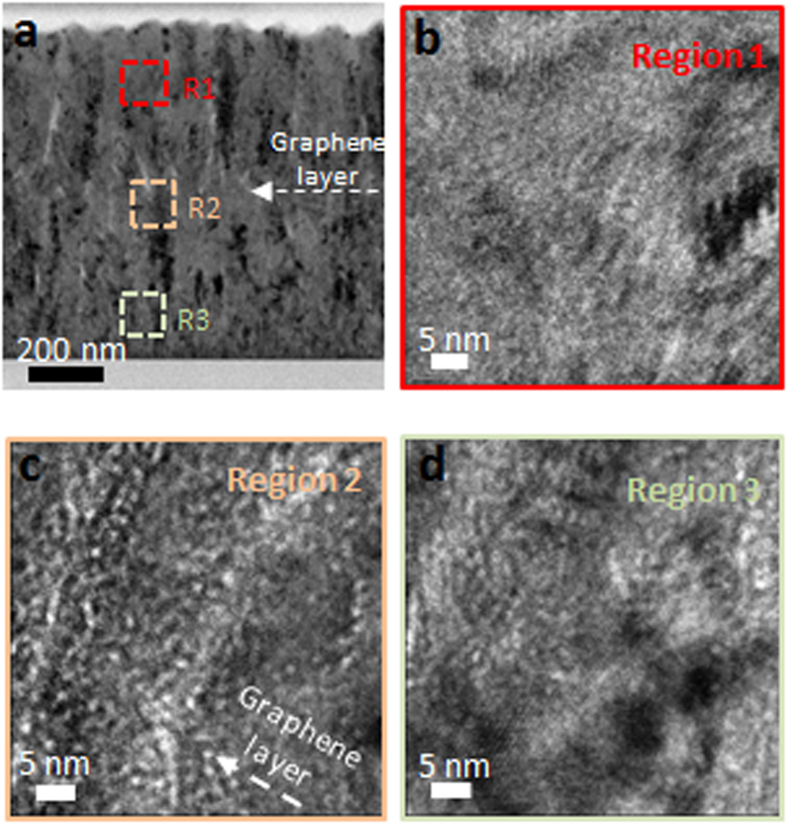
(**a**) TEM analysis of the V-graphene nanolayer after He^+^ irradiation at 120 keV for V-graphene with 300 nm repeat layer. High magnification images are taken using 400 nm under-focus condition at (**b**) the top layer, (**c**) the graphene interface, and (**d**) the bottom layer. White dots are indicative of He bubbles, which are concentrated near the graphene interface shown in (**c**).

As outlined above, He bubble formations are of concern in maintaining the mechanical stability in nuclear structural materials. He is known to rapidly combine with vacancy clusters to form bubbles that can migrate and agglomerate to result in intergranular failure by embrittlement and crck formation^41^,^42^,^43^. However, atomistic simulations on Cu-Nb nanolayers showed that the interfaces can act as sinks for high concentrations of vacancies due to the lower formation energies of vacancies at the Cu-Nb interfaces that can lead to lower density of He bubbles in Cu-Nb nanolayers in comparison to that in bulk Cu or Nb^29^,^44^,^45^,^46^. The V-graphene nanolayers are expected to also be excellent at terminating He gas migration in comparison to the nanolayers composed of only metal layers due to the impermeability of the He gas through the graphene layer^35^. Cross-section TEM images of V-graphene with 300 nm repeat layer spacing was taken at different locations as marked in Fig. 3 using 400 nm under-focus condition. He bubbles were observed at the top V surface region as shown in Fig. 3b, but most He bubbles were concentrated at the graphene interface that are shown as white dots in Fig. 3c. The graphene interface hindered the He bubbles from migrating and agglomerating to larger sizes, which can potentially penetrate through thickness of the layers to result in blister. It should be noted again that the chosen irradiation energy is sufficient for He ions to have penetrated through thickness of the nanolayers, but the graphene interface has effectively stopped migration of He bubbles.

### Role of graphene in reducing radiation induced hardening

The increase in yield strength after He implantation is commonly accepted to be due to the formation of radiation induced crystalline defects as well as He bubbles^22^,^23^,^24^,^47^,^48^. Our results indicate that the flow stress at 5% strain for V-graphene with 300 nm and 110 nm repeat layer spacing exposed to He^+^ irradiation were increased by 40% and 25%, respectively while pure V after irradiation resulted in increase in flow stress of 88%. Significant reduction in radiation hardening in V-graphene nanolayers is expected to be due to reduction in He bubble formation, as well as the ability of the V-graphene interface to absorb the crystalline defects. The total radiation hardening can be expressed as


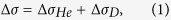


where 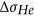 is the radiation induced hardening in the presence of He bubbles and 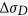 is from other crystalline defects. Each of these contributions is individually considered for the case of V-graphene nanolayers.

Radiation induced hardening due to presence of He bubbles (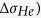 can be calculated from Friedel–Kroupa–Hirsch (FKH) model for weak obstacles^49^,^50^, where the increase in yield strength is given by


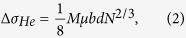


where *M* is Taylor factor, 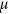 is the shear modulus, *b* is the Burgers vector, *d* is bubble diameter and *N* is bubble density. For the measured He bubble size from TEM images for V-graphene with 110 nm repeat layer spacing, the radiation induced hardening is calculated to be 260 MPa for typical values of *M*, 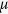, b for V metal. The calculated value is smaller than that of the experimentally measured radiation induced hardening of 1200 MPa. Therefore, 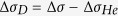 is calculated to 940 MPa, which can be attributed to radiation induced hardening from other crystalline defects that are introduced from collision cascade. Orowan’s model can now be used to determine the approximate length scale for the existing pinning points within the V matrix.


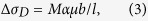


where 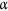 is the barrier strength, 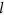 is average spacing between obstacles then given by 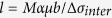 = 10.1 nm. Similar calculations of 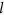 for V-graphene with 300 nm repeat layer spacing and pure V are 7.6 nm and 4.8 nm, respectively. This analysis indicates that the length scale for separation distance between the crystalline defects is the largest for the case with V-graphene with 110 nm spacing, which is indicative of the V-graphene interface effectively absorbing the crystalline defects from the V matrix to minimize radiation induced hardening. In the work by Zhang *et al.*, radiation induced hardening variation of Cu-V with 100 nm spacing under total ion fluence 6 × 10^20^ ions/m^2^ was reported to be 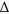
0.7 GPa, which again confirms that our V-graphene with 100 nm spacing is significantly more efficient with radiation induced hardening of 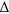
1.2 GPa under higher total ion fluence of 1 × 10^22^ ions/m^2^.

### Molecular dynamics simulations: Analysis

**Figure 4:**
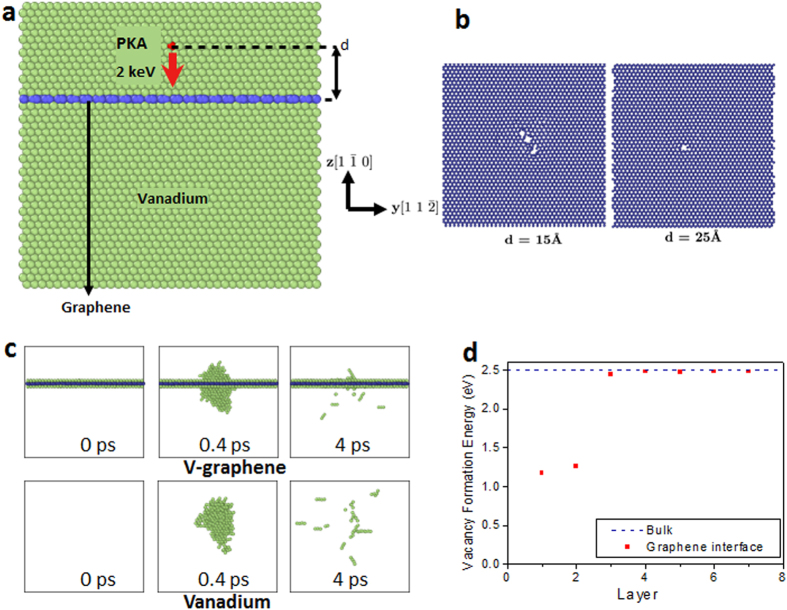
Molecular dynamics simulations of the knock-on event. (**a**) Schematic of simulation. (**b**) Damage on graphene after the knock-on event. The damage on graphene reduces as the distance to PKA increases because large portion of the energy is released by the vacancy-interstitial formation as well as local heating in lattice. (**c**) The collision cascade after the knock-on event for d = 15 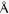. The amount of cascade is significantly reduced by the graphene layer. Significantly more defects remain in the pure V. Atoms with high potential energy (above −4.4 eV) are visualized selectively. (**d**) The formation energies of vacancies are significantly lower at the graphene interface than in the bulk lattice of V. The migrated vacancies can then be annihilated with the crowdions to result in self-healing of radiation induced defects.

The role of graphene in enhancing the radiation tolerance was investigated by employing molecular dynamics (MD) simulations on a model system of pure V and V-graphene nanolayers. We model the radiation event as a collision of a primary knock-on atom (PKA) with 2 keV (See Fig. 4a). Because the interaction between V and graphene is van der Waal’s type, there are no preexisting defects or misfit dislocations at the interface. The detailed interface structure and defect-interface characteristics before the collision is available in the Supplementary Information Note 3, 4. After the initial explosive collision cascade event, numerous vacancy and interstitial pairs are created. For the body-centered cubic (bcc) crystal such as V, a special type of delocalized mobile interstitial, called crowdion, is formed which can move along the [111] direction quickly without large thermal activation. Crowdions can be recognized as small chains of 3-4 high energy atoms in the Fig. 4c and the Supplementary Movie 2 and 3. The MD simulation results indicate that the radiation induced of V-graphene nanolayers is less severe than that of pure V since the number of remaining point defects from the collision cascade becomes significantly smaller in the presence of graphene layers, as compared in Fig. 4.

One of the main reasons for the reduction in crystalline defects in the presence of the graphene interface is due to the reduction in the vacancy-interstitial formation. The knock-on energy from the collision cascade is typically relaxed through local heating of the crystal lattice as well as by forming vacancy-interstitial pairs, but a large portion of the energy in the case of V-graphene nanolayers is consumed to break the covalent bonds in graphene due to the high atomization energy (7.4 eV) of the graphene. Thus, significant damage of graphene is observed when the knock-on event occurs close to graphene layers (see Fig. 4b), and this results in reduction of the size of the collision cascade in comparison to that in pure V.

Second reason for less defect formations is due to the V-graphene interface acting as an efficient sink for the interstitial defects or crowdions. Crowdions move along the [111] directions until they encounter the V-graphene interface, and form adatoms between the graphene and V. Vacancies also diffuse to the V-graphene interface and the arrived vacancies are then trapped at the interface because the vacancy formation energy is smaller at the graphene interface (1.2 eV) than in the bulk lattice (2.5 eV) (see Fig. 4d). The vacancy migration mechanism due to its lower formation energy at the interface was also observed in the atomistic modeling of Cu/Nb interface by Misra *et al.*^24^. Using the Fick’s first law, one can estimate the time scale (*t*) for the vacancy to arrive at the interface as


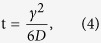



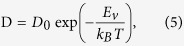


where 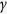 is diffusion distance assumed to be half of the layer spacing (300 nm → 150 nm, 110 nm → 55 nm) and D is the vacancy diffusion coefficient, 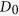 is Debye frequency of 10^13 ^Hz, 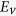 is vacancy jump energy barrier calculated from nudged elestic band calculations^51^ as 0.187 eV, 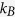 is Boltzmann constant and T is absolute temperature. The timescales for the vacancy to arrive at the graphene interface for V-graphene with 110 nm and 300 nm repeated layer spacings are 3.27 × 10^4^ s and 2.43 × 10^5^ s, respectively, at room temperature. However, during the ion irradiation testing and FIB processing to fabricate pillars for compression testing, the actual temperature can increase up to ~200 °C that could then enable vacancies to reach the interface in just a 0.26 seconds for V-graphene with 110 nm repeated layer spacing. Therefore it is expected that the interstitials in the form of crowdion and vacancies gathered at the interface to annihilate near the graphene interfaces. Higher density of the V-graphene interfaces (*i.e.* small repeat layer spacings) results in more effective annihilation of interstitials and vacancies, thereby increasing the self-healing ability of the nanolayers. Our analysis is consistent with the experimental observation in which the V-graphene with 110 nm repeat layer spacing showed significantly smaller radiation induced hardening than in 300 nm repeat layer spacing specimen. The self-healing ability as confirmed from MD simulations is expected to be one of the major causes for enhanced radiation tolerance in V-graphene nanolayers, while suppression of He bubbles at the graphene interface also suppress the brittle failure.

### Summary

In this study, we developed and analyzed the radiation resistance V-graphene nanolayered composites with varying repeat layer spacings of 110 nm, 300 nm and compared the results to those of pure V. He^+^ irradiation with dosage of 13.5 dpa revealed that the V-graphene nanolayers had reduced formations of radiation induced crystalline defects compared with pure V, which agreed with the nanopillar compression results of irradiated specimen V-graphene showing reduction in radiation induced hardening and suppression of brittle failure. *In-situ* SEM compression tests showed that the graphene interface can hinder the crack propagation thus helping to suppress the brittle failure. The structural material degradation due to He bubble formations was shown to be significantly reduced in V-graphene nanolayers due to the impermeability of He through the graphene layer that prevents agglomeration of He gas into large bubbles. Orowan’s model was then used to determine the approximate length scale for the pinning points from the presence of radiation induced defects that indicated less accrual of defects in the V-graphene layers. Finally, MD simulations confirmed that the cause for less crystalline defects in V-graphene layers is due to the graphene interface being able to absorb the crystalline defects that are introduced from collision cascade. Therefore, inclusion of graphene in the form of V-graphene nanolayers can not only result in initially high strength material, but the graphene can self-heal the crystalline defects that are introduced during irradiation as well as terminating migration of He bubbles to result in extremely radiation resistance material.

## Methods

### Synthesis of V-graphene nanolayers

The Si/SiO_2_ (625 μm/300 nm) substrate was chosen for deposition of the V-graphene nanolayers. Vanadium nanolayers were first deposited using radio frequency (RF) sputtering under vacuum at 5 × 10^−6^ Torr with Ar gas of 5 mTorr at 10 sccm, and RF power of 200 W was used. After deposition of the V layer, graphene was fabricated by chemical vapor deposition (CVD) and then transferred on to Si/SiO_2_/V substrate. By repeating the above steps, nanocrystalline V-graphene nanolayers composites were synthesized with 110 nm and 300 nm repeat layer spacings, while keeping the total thickness to be the same at ~600 nm.

### Nanopillar compression testing

To test the mechanical properties of the V-graphene nanolayers composites, the Quanta 3D FEG Focused ion beam (FIB) milling was utilized to fabricate V-graphene nanopillars with diameter of 250 nm and height of 600 nm. The pillar compression tests were performed by the Hysitron Tbi-750 (Minneapolis, MN) nanoindentation system with a 10 μm diameter flat-ended cube corner tip. All pillars were compressed over 15% total strain in a displacement control feedback mode (DC mode) using the Hysitron quasi-static indentation transducer with nominal engineering strain rate of 0.002 s^−1^. The load-displacement data from compression test were analyzed with the conventional constant volume and homogeneous deformation assumption model to calculate the true stress-true strain curves^52^. *In-situ* SEM nanopillar compression tests were also conducted to confirm the deformation behavior of the nanopillars using a Hysitron Picoindenter (PI-95) in a Quanta 3D FEG FIB. Same sized nanopillars were compressed under *in-situ* SEM at a nominal constant strain rate of 0.002 s^−1^.

### He^+^ irradiation

To evaluate the radiation resistance of V-graphene nanolayers, He^+^ ion irradiation was performed at Kyungjoo Korea Multi-Purpose Accelerator Complex (KAERI) facility at 120 keV with beam current of 1.2 mA and dosage of 1 × 10^18^/cm^2^ at room temperature. The SRIM calculations indicate (state the simulation conditions and assumptions) that this irradiation condition is sufficient to reach through thickness of the V layers down close to the substrate interface. The benefits of ion irradiation in probing the radiation resistance of materials rather than using neutron irradiations were previously reported in the work by Kiener *et al.*^53^.

## Additional Information

**How to cite this article**: Kim, Y. *et al.* Radiation Resistant Vanadium-Graphene Nanolayered Composite. *Sci. Rep.* **6**, 24785; doi: 10.1038/srep24785 (2016).

## Supplementary Material

Supplementary Information

Supplementary Movie 1

Supplementary Movie 2

Supplementary Movie 3
