# Supplementary material for: Radiation Resistant Vanadium-Graphene Nanolayered Composite
Source: Sci Rep. 2016 Apr 21;6:24785. doi: 10.1038/srep24785 (PMC4838849; doi:10.1038/srep24785)
Supplement: Supplementary Information [file srep24785-s1.pdf]

Supplementary Information

Radiation Resistant Vanadium-Graphene Nanolayered Composite

Youbin Kim[1], Jinwook Baek[2], Sunghwan Kim[3], Sangmin Kim[1], Seunghwa Ryu[3], Seokwoo Jeon[2], Seung Min Han[1*]

[1] Graduate School of Energy, Environment, Water and Sustainability, Korea Advanced Institute of Science & Technology, Daejeon 305-338, Korea

[2] Department of Materials Science and Engineering and Graphene Research Center of KI for the NanoCentury, Korea Advanced Institute of Science & Technology, Daejeon 305-338, Korea

[3] Department of Mechanical Engineering, Korea Advanced Institute of Science & Technology, Daejeon 305-338, Korea

*To whom correspondence should be addressed. E-mail: S.M.H (smhan01@kaist.ac.kr)

Supplementary Note 1. Graphene layer analysis with XPS

Single layer of graphene was transfered on the V thin film to anaylze the formation of vanadium carbide at the graphene and vanadium interface using X-ray photoelectron spectroscopy (XPS). Binding energy spectrum of V-graphene specimen was recorded with K-alpha XPS under the condition of 500 eV, 0.05 nm/s with Ar ion gun. The depth profile of the carbon Cls spectrum as shown in the Supplementary Fig. 1 indicate that the graphene sp^2 C-C bonding are present at 284.8 eV and the PMMA residue is responsible for the weak peak at 289 eV. Forthermore, any formations of vanadium carbide (VC) is not detected which has binding energy of 282 eV. According to the phase diagram, VC should not form at room temperature but is observed at higher temperatures of ~800℃.[1]

Supplementary Note 2. Compression testing results for before and after irradiation.

The nanopillars were fabricated from pure V and V-graphene nanolayers with 110 nm and 300 nm repeat layer spacings (Supplementary Fig. 2a and c) for compression testing. The SEM images of the deformed nanopillars shown in (Supplementary Fig. 2b and d) indicate that both pure V and V-graphene nanolayers plastically deformed up to strain of 25% without any brittle failure. The pure V nanopillars before and after He^+ irradiation showed strikingly different deformation behavior in that a clear embrittlement was observed in the irradiated specimen as shown in Fig. 2d. V-graphene nanopillars before He^+ irradiation showed no signs of embrittlement while irradiation resulted in crack formation at the top layer, which was stopped by the graphene interface as shown in Fig. 2e.

Supplementary Note 3. Molecular dynamics simulations: Method

MD simulations were performed using the Large-scale Atomic/Molecular Massively Parallel Simulator (LAMMPS)[2]. The embedded-atom-method (EAM) interatomic potential, developed by Mendelev *el al.*, was used to properly describe the interatomic interactions in V[3]. ZBL potential is augmented at short distances to fix the soft core of EAM potential[4]. To describe the interactions between carbon atoms in graphene, adaptive intermolecular reactive empirical bond order (AIREBO) potential was used[5]. The interaction between V and carbon atoms are modelled as 12-6 Lennard-Jones (LJ) type of van der Waal's interaction with ϵ = 0.024 eV and σ = 2.073 Å, optimized by *ab initio* calculation from the Vienna *Ab initio* Simulation Package (VASP) [6].

We prepared two samples, pure V and V-graphene composite, to investigate the role of graphene in radiation damage. The sizes of pure V cell and V-graphene cell are 93.28 × 94.93 × 87.55 $Å^3$ (58,968 atoms) and 93.50 × 95.17 × 89.82 $Å^3$ (62,556 atoms), respectively. For the V-graphene composite, graphene is inserted between two (110) V surfaces (the lowest surface). To minimize the in-plane misfit strain, the vanadium is constructed with 18 [111] × 13 [$11\bar{2}$] vanadium unit cell along x and y direction, while graphene has armchair edge in x direction with length $39\sqrt{3}a$ and zigzag edge with $69a$ long in y direction, with a = 1.42 Å. Resultantly, 0.24% and 0.25% in-plane misfit strains are imposed at the V-graphene interface along each direction.

The knock-on event is described by assigning 2 keV kinetic energy on PKA, and following dynamics are traced with the NVE ensemble. The default value of the time step is chosen as 0.1 fs, while it is dynamically adjusted to limit the maximum displacement in one time step within 0.001 Å.

Supplementary Note 4. Defects-interface characteristics

The interaction energy between the V (110) substrate and a graphene sheet is about 0.4 eV/atom as shown in Supplementary Fig. 3 that is much smaller than V-V interaction and graphene covalent interaction energy of 5.3 eV and 7.4 eV, respectively. Due to the weak van der Waals interaction between V and graphene, the misfit dislocation structures that are typically formed at the metal-metal interface[7-10] is not observed in our metal-graphene system. If there exist dislocations at interface, it should be the blocked gliding dislocations during plastic deformation.[11]

Our MD simulation results on V-graphene interfaces indicated that the interface can acts as sink for vacancy- interstitial pairs. The interstitials move along the [111] directions and the vacancies diffuse to the V-graphene interface because the vacancy formation energy at interface is lower one-half times than bulk V. Therefore, the V-graphene interface can potentially have self-healing ability to absorb the point defects.

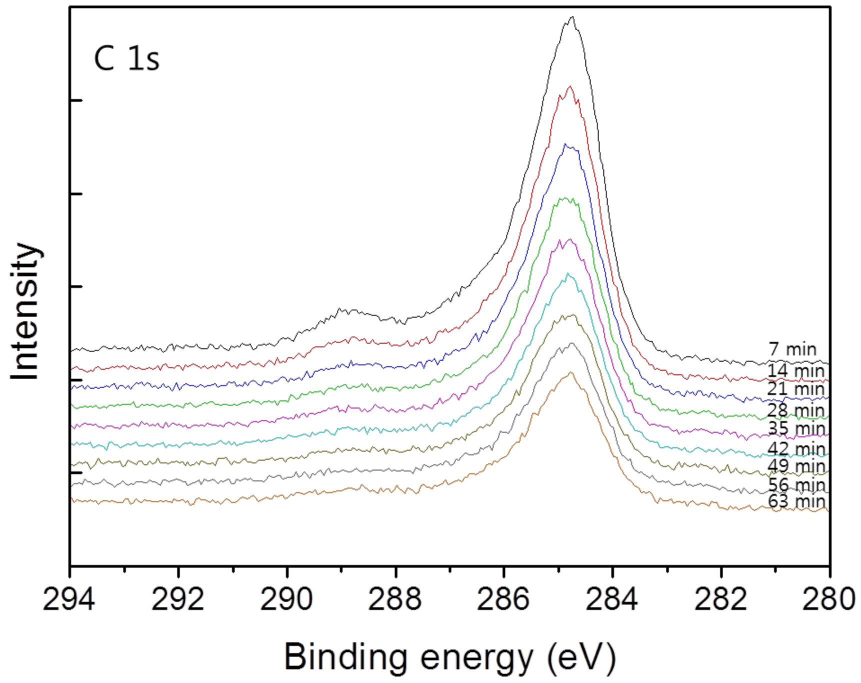

Supplementary Figure 1 XPS spactrum of vanadium-graphene thin film indicated that the formation of sp^2 C-C bonding of graphene where C 1s peaks appear at 284.8 eV. No VC formation between graphene and vandium was observed.

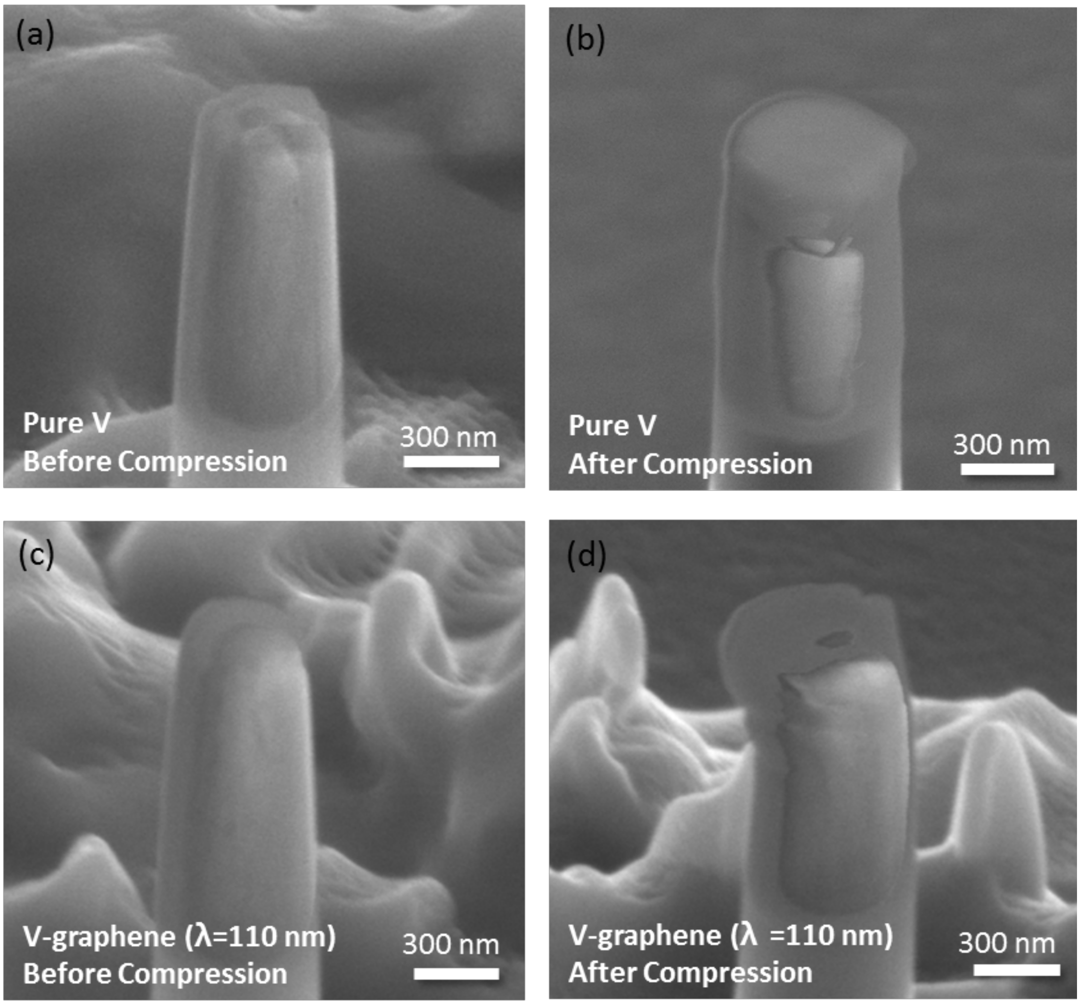

Supplementary Figure 2 SEM images of nanopillars (a) before and (b) after compression testing for pure V, and V-graphene with 110 nm repeat layer spacing (c-d).

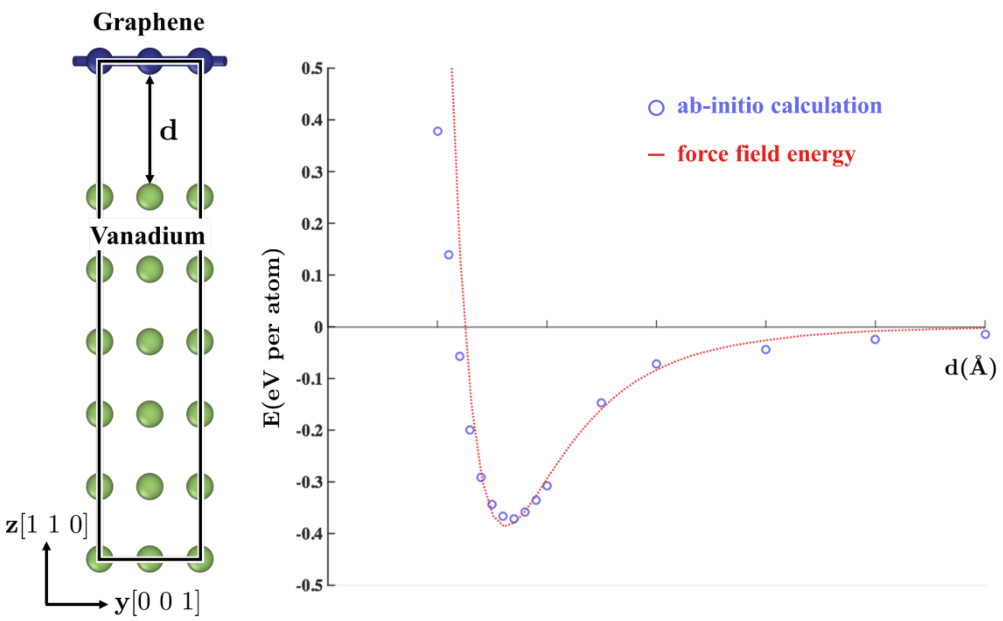

Supplementary Figure 3 Interaction energy curves for V-graphene interface. The interaction energy between the V (110) substrate and a graphene sheet is computed by using the local density approximation (LDA) with the van der Waals correction. The Lennard-Jone parameters are optimized to fit the interaction energy from the *ab initio* calculation. Due to the lattice mismatch of V-graphene interface, the unit cell of graphene is adjusted to the size of V unit cell.[1]

Supplementary references

[1] G. Bauer, *et al.*, *Ullmann's encyclopedia of industrial chemistry* (1996).
[2] S. Plimpton, *J. Com. Phy*. **117**, 1 (1995).
[3] M. I. Mendelev, *et al.*, *Phy. Rev. B*. **76**, 214105 (2007)
[4] C. Björkas, *et al.*, *Nuclear Instruments and .Methods in Physics Research Section B: Beam Interactions with Materials and Atoms*. **267**, 3204 (2009).
[5] S. J. Stuart, *et al.*, *J. Chem. Phy*. **112**, 6472 (2000).
[6] G. Kresse, *et al.*, *Phy. Rev. B*. **54**, 11169 (1996).
[7] S.Shao, *et al.*, *Sci. Rep*. **3**, 2448 (2013)
[8] S.Shao, *et al.*, *J. Appl. Phy*. **116**, 023508 (2014)
[9] S.Shao, *et al.*, *Acta. Mat*. **86**, 331 (2015)
[10] S.Shao, *et al.*, *Acta. Mat*. **98**, 206 (2015)
[11] Y. Kim, *et al.*, *Nat. Com*, **4**, 3114 (2013)

Supplementary Video 1. In-situ SEM compression test of V-graphene. In-situ SEM compression testing of V-graphene with 110 nm repeat layer spacing shows that the crack propagation was suppressed by the graphene interface.

Supplementary Video 2. Molecular simulation of Vanadium. Crowdions can be recognized as small chains of 3-4 high energy atoms in Vanadium.

Supplementary Video 3. Molecular simulation of V-graphene. Remaining point defects from the collision cascade becomes significantly smaller in V-graphene than in pure V.
